# Ag Nanoparticle-Decorated Oxide Coatings Formed via Plasma Electrolytic Oxidation on ZrNb Alloy

**DOI:** 10.3390/ma12223742

**Published:** 2019-11-13

**Authors:** Oleksandr Oleshko, Volodymyr Deineka V, Yevgeniia Husak, Viktoriia Korniienko, Oleg Mishchenko, Viktoriia Holubnycha, Marcin Pisarek, Joanna Michalska, Alicja Kazek-Kęsik, Agata Jakóbik-Kolon, Wojciech Simka, Maksym Pogorielov

**Affiliations:** 1 Center of Collective Use of Scientific Equipment, Sumy State University, 40018 Sumy, Ukraine; oleshkosanya007@gmail.com (O.O.); evgenia.husak@gmail.com (Y.H.); vicorn77g@gmail.com (V.K.); golubnichiy@ukr.net (V.H.); 2 Osteoplant Research and Development, 39-200 Dębica, Poland; dr.mischenko@icloud.com; 3 Institute of Physical Chemistry, 01-224 Warsaw, Poland; mpisarek@ichf.edu.pl; 4 Faculty of Chemistry, Silesian University of Technology, 44-100 Gliwice, Poland; joanna.k.michalska@polsl.pl (J.M.); alicja.kazek-kesik@polsl.pl (A.K.-K.); agata.jakobik-kolon@polsl.pl (A.J.-K.)

**Keywords:** plasma electrolytic oxidation, dental implant, Ag nanoparticles (AgNPs), biocompatibility, bacterial adhesion

## Abstract

Plasma electrolytic oxidation (PEO) can provide an ideal surface for osteogenic cell attachment and proliferation with further successful osteointegration. However, the same surface is attractive for bacteria due to similar mechanisms of adhesion in prokaryotic and eukaryotic cells. This issue requires the application of additional surface treatments for effective prevention of postoperative infectious complications. In the present work, ZrNb alloy was treated in a Ca-P solution with Ag nanoparticles (AgNPs) for the development of a new oxide layer that hosted osteogenic cells and prevented bacterial adhesion. For the PEO, 0.5 M Ca(H_2_PO_2_)_2_ solution with 264 mg L^−1^ of round-shaped AgNPs was used. Scanning electron microscopy with energy-dispersive x-ray and x-ray photoelectron spectroscopy were used for morphology and chemical analysis of the obtained samples; the SBF immersion test, bacteria adhesion test, and osteoblast cell culture were used for biological investigation. PEO in a Ca-P bath with AgNPs provides the formation of a mesoporous oxide layer that supports osteoblast cell adhesion and proliferation. Additionally, the obtained surface with incorporated Ag prevents bacterial adhesion in the first 6 h after immersion in a pathogen suspension, which can be an effective approach to prevent infectious complications after implantation.

## 1. Introduction

Incomplete osteointegration and microbial infection represent major contributors to implant failure. Microbial populations use cell attachment to solid substrates to survive, forming biofilms [1]. Strategies for bone and dental implant development have focused on surface modification to improve implant osteointegration and reduce bacterial infection. The development of multifunctional strategies that promote osteointegration while mitigating bacterial colonization is clearly important because both effects are necessary to ensure optimal, long-term functionality of medical implants. However, the adhesion mechanisms of eukaryotic and prokaryotic cells are similar, and this is a critical consideration in multifunctional surface development.

Plasma electrolytic oxidation (PEO) is well known as a promising method for dental and orthopaedic implant modification due to its ability to improve corrosion and wear resistance [2], biocompatibility, and bioactivity [3]. This technique provides an excellent solution for bone cell attachment and proliferation and shows a good in vivo response. However, at the same time, there is much evidence that the PEO surface can provide support for bacterial cell attachment, requiring the application of antibacterial agents on the implant surface.

Silver (Ag) is well-known ion with strong antibacterial effects that have been used since ancient time, and the application of nanostructural technologies can significantly increase these effects. Ag is widely used as an antibacterial coating due to its excellent antimicrobial properties and resistance against a wide variety of microbes, including gram-positive and gram-negative bacteria [4]. There have been some studies about the application of Ag nanoparticles (AgNPs) in the PEO of Ti implants. Necula et al. [5] incorporated AgNPs with an average size of 37 ± 6 nm in a TiO_2_ coating through PEO and showed total elimination of MRSA bacteria after culturing for 24 h. Hengel et al. [6] provided a PEO coating with AgNPs on porous implants and showed a two-fold larger zone of inhibition than that of the untreated control, while no signs of cytotoxicity were observed in human mesenchymal stem cells. But antibacterial coatings with high concentrations of Ag have been found to exhibit cytotoxicity and inhibit osteointegration. Necula et al. [7] compared TiO_2_ coatings with several concentrations of AgNPs and found that the concentration of 0.3 mg L^−1^ Ag had no toxic effect on the human osteoblastic cell line (SV-HFO), while 3.0 mg L^−1^ was extremely cytotoxic. In a similar study, Shin et al. found no cytotoxic response at low AgNP concentrations (0.1 g L^−1^) in the MC3T3-E1 cell line, but increasing concentrations (0.3 and 0.5 g L^−1^) had a negative impact on cell proliferation [8]. Zhang et al. [9,10] found that PEO surfaces with a Ag content of 1.36 wt % and above were reported to be cytotoxic.

Most of the implants used in orthopedics and dentistry are made from Ti-6Al-4V alloy [11] with higher elastic stiffness and a relatively high elastic modulus (110 GPa) that in 4–6 time higher the cortical bone (30 GPa). It is well known that decreasing of elastic modulus induces a better stress transfer along the bone–implant interface and could limit the crestal bone loss. Moduli mismatch could lead to excessive micro-motion between implants and bone, which prohibits bone formation and contrarily facilitates fibrous tissue ingrowth [12,13]. Additionally, Ti-6Al-4V alloy can release Al and V ions during corrosion (after long term implantation) that should make an adverse tissue reaction. New metastable beta Ti-based alloys (contained nontoxic elements such as Nb, Ta, Mo, and Zr) can partially solve this problem due to adjusted chemical compositions that display a reversible stress-induced martensitic transformation that leads to a superelastic effect (high recoverable strain) and a very significant reduction of the apparent elastic modulus [14]. ZrNb alloys are a new promising solution to produce low-modulus orthopaedics and dental implants [15]. They have great potential due to the ability of PEO to improve the appropriate mechanical properties [7,16]. However, there is a lack of data about PEO surface functionalization of Zr alloys with AgNPs for the development of antibacterial coatings.

The aim of the current research was to evaluate the structural and chemical properties of ZrNb alloy surfaces and their antibacterial and cytotoxicity activities after PEO in a Ca-P solution with the addition of AgNPs.

## 2. Materials and Methods 

### 2.1. Materials 

A ZrNb alloy (Zr-2.5 wt % Nb) was obtained from Osteoplant R&D (Dębica, Poland); 6 mm diameter cylindrical samples with a height of 6 mm were prepared for all investigations. All chemicals were purchased from Sigma-Aldrich (Darmstadt, Germany) and used as received: silver nitrate (AgNO_3_, 98%), poly(N-vinylpyrrolidone) (PVP, M_w_ = 40,000, K-25), sodium phosphinate monohydrate (SPM, NaH_2_PO_2_⋅H_2_O, 95%), ethylene glycol (EG, C_2_H_6_O_2_, 90%), isopropanol (C_3_H_8_O, 90%), and calcium hypophosphite (Ca(H_2_PO_2_)_2_).

*Staphylococcus aureus* B 918, obtained from the National Collection of Microorganisms (D.K. Zabolotny Institute of Microbiology and Virology, Kiev, Ukraine), was used in the experiment. All bacteriological media were purchased from HiMedia (Maharashtra, India) and Alamar blue was from Invitrogen (Carlsbad, CA, USA). For the cell culture study, all media and reagents were purchased from Gibco®, Gaithersburg, MD, USA. Human dermal fibroblasts (HDF) and human osteoblasts (HO) were obtained from the collection of Sumy State University, Sumy, Ukraine.

### 2.2. Synthesis of Ag Nanoparticles

First, 10 mL of EG was heated to 160 °C for 30 min, and 5 mL of 0.1 M PVP/EG solution was added to the heated EG solution. Afterwards, 5 mL of 0.2 M AgNO_3_/EG solution was added dropwise to the reaction flask, and the mixture was magnetically stirred for 1 h. Once the reaction was finished, 10 mL of isopropanol was added to terminate the reaction. The sample was washed by repeated centrifugation at 4000 rpm for 10 min with re-dispersion in isopropanol. The AgNP synthesis was described elsewhere [17]. We have calculated reaction time for Ag growth from nucleation event start point to termination by adding IPA (reaction temperature decreased below the growth threshold). Some aliquots were taken during the reaction event by pulling out the reaction mixture with a syringe filled with IPA.

### 2.3. Plasma Electrolytic Oxidation

All ZrNb alloy samples were rinsed with distilled water and ultrasonically cleaned in deionized water and 2-propanol for 5 min. The samples were subjected to anodization in an electrolytic bath containing Ca(H_2_PO_2_)_2_ (0.5 M) and silver nanoparticles (264 mg L^−1^). The anodic oxidation was performed under a constant current density of 0.1 A cm^−2^ and up to a final voltage of 450 V for 5 min. The surface area of the face that was modified was equivalent to 0.89 cm^2^. The anodized specimens were rinsed with distilled water and deionized water for 5 min.

A DC power supply (PWR 800H, Kikusui, Japan) was used throughout these treatments. The process was performed in a water-cooled electrolyzer cell with a titanium mesh cathode and magnetic stirrer. The ZrNb alloy served as the anode.

### 2.4. Surface Analysis

SEM investigations were used to assess the sample morphology and chemical composition after the PEO process. Tescan (Brno – Kohoutovice, Czech Republic) and Phenom Pro X (Eindhoven, The Netherlands) scanning electron microscopes with an EDX system were used for the observation and analysis of the samples after the PEO process. AgNPs were observed with a JEOL SEM system (Tokyo, Japan).

The chemical composition and chemical state of the sample were characterized by X-ray photoelectron spectroscopy (XPS). For this purpose, a PHI 5000 VersaProbe (ULVAC-PHI) spectrometer (Chigasaki, Japan) was used. The XPS spectra were obtained using Al_Kα_ (hν = 1486.6 eV, 25 W) monochromatic radiation as a source. The survey and high-resolution spectra were collected with a hemispherical analyser at constant pass energies of 117.4 and 23.5 eV, respectively. The background was corrected using the Shirley model to obtain the XPS signal intensity. An asymmetric Gaussian/Lorentzian function at a constant ratio of G/L = 0.35 was used for the deconvolution procedure. The determined peak positions were corrected in relation to that of adventitious carbon C1s at 284.8 eV. Avantage Surface Chemical Analysis software (ver. 5.9904) was used for the data processing.

A roughness of the samples was measured with the use of Mitutoyo Surftest SJ-301 (Kawasaki, Japan). Contact angle measurements were done with use of a OCA 15 EC (Data Physics, Filderstadt, Germany) instrument.

### 2.5. Bacterial Adhesion Test

The adhesive properties of the differently processed samples were assessed using a gram-positive bacterium (*S. aureus*, strain B 918). The bacterial strain grown on nutrient agar at 37 °C for 24 h was suspended in a saline solution (0.9%, *w*/*v* NaCl) and re-suspended to a final density of 1 × 10^8^ colony forming units (CFUs) mL^−1^ (8 log CFU) in nutrient broth using McFarland standards.

The discs were incubated horizontally with 2.0 mL of the bacterial suspension in static conditions in a 24-well plate at 37 °C for 2, 4, 6, and 24 h. Then, the specimens were removed with sterile forceps and washed with 2.0 mL of sterile physiological saline three times to remove unadhered bacteria. Next, the discs were placed in sterile tubes with 1.0 mL of sterile saline solution and sonicated for 1 min by using an ultrasonic bath (B3500S-MT, Bransone Ultrasonics Co., Shanghai, China) to remove adherent bacteria from the surfaces of the specimens. Following this step, we conducted determinations of the colony count at each incubation time point by using the streak plate technique, cultivating 10-μL aliquots of saline solution from the sonicated tubes on the solid media for 24 h. The wells containing the discs and tested samples in growth medium without the bacterial inoculates were used as a control. As positive controls, non-treated polished ZrNb discs and ZrNb-PEO without AgNPs were used. All experiments were conducted in triplicate.

### 2.6. In Vitro Evaluation of Ion Release from Coatings

The anodized samples were placed separately in Falcon tubes, each with 20 mL of Ringer solution. The samples were shaken at 60 rpm and incubated at 37 °C (incubator POL-EKO, Wodzisław Śląski, Poland) for up to 6 weeks. After each 2-week period, the content of niobium, phosphorus, zirconium, calcium, and silver released into the Ringer solution was determined using inductively coupled plasma atomic emission spectrometry (ICP-AES). A Varian 710-ES spectrometer (Santa Clara, USA). equipped with a OneNeb nebulizer was utilized. The parameters were as follows: RF power 1.0 kW, plasma flow 15 L min^−1^, auxiliary flow 1.5 L min^−1^, nebulizer pressure 210 kPa, pump rate 15 rpm; emission lines of Nb:λ = 295.088 and 294.154 nm, P:λ = 213.618, Zr:λ = 343.823 and 327.307 nm, Ca:λ = 317.933 and 422.673 nm, and Ag:λ = 328.068 and 338.289 nm. The calibration curve method was applied. The calibration curves were prepared using a matrix (Ringer solution) at the same concentration as that of the samples. Single element standard solutions of 1 and 10 mg mL^−1^ (zirconium) supplied by Merck Millipore (Darmstadt, Germany) were used. Deionized water was prepared using a Millipore Elix 10 system (Darmstadt, Germany). The obtained results are the average of concentrations obtained for all used analytical lines with the standard deviation not exceeding 1.5%.

### 2.7. Cell Culture

The cells were grown in 75 cm^2^ tissue culture flasks under standard culture conditions of 5% CO_2_ and humidified air at 37 °C with medium renewal for every 2–3 d. Dulbecco’s modified Eagle medium/nutrient mixture F-12 (DMEM/F-12) with L-glutamine was used, containing 100 units mL^−1^ penicillin, 100 µg mL^−1^ streptomycin, 2.5 µg mL^−1^ amphotericin B, 10% foetal bovine serum, and 1.0 ng mL^−1^ bFGF. After the medium was removed, osteoblasts were seeded on each sample and in the positive control wells at a cell density of 2 × 10^4^ cells per well. Cell adhesion at 24 h and cell proliferation on samples were assessed by the Alamar blue colorimetric assay, which is used to measure cell viability. Alamar blue (Invitrogen) was added in an amount equal to 10% of the volume to each well. As a negative control, Alamar blue solution was added to the medium without cells. As a positive control, Alamar blue solution was added to the medium of wells that contained only cells without samples (TCP control) and with non-modified polished ZrNb alloy. The plates were incubated for 4 h at 37 °C in the dark. The medium was transferred to another 96-well plate, and the absorbance was measured using a Multiskan FC (Thermo Fisher Scientific, Waltham, MA, USA) plate reader at wavelengths of 570 and 600 nm. The cells were quantified at different time intervals: 1, 3, and 7 d. All experiments were repeated 3 times.

### 2.8. Statistic

Data was expressed as means ± standard deviation. Student’s t-test on unpaired data was used to assess the statistical significance of the difference. Statistical significance was assumed at a confidence level of 95% (*p* < 0.05).

## 3. Results and Discussion

**Figure 1:**
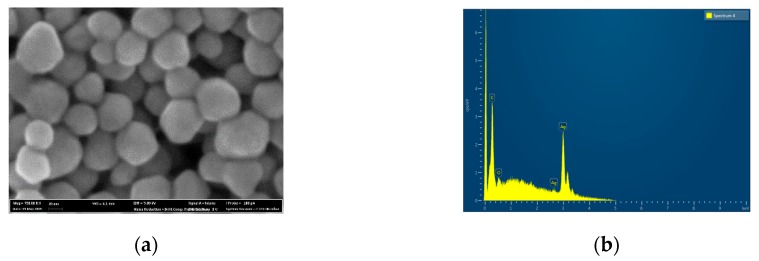
SEM image (**a**) and EDX spectra (**b**) of Ag nanoparticles (AgNPs).

**Figure 2:**
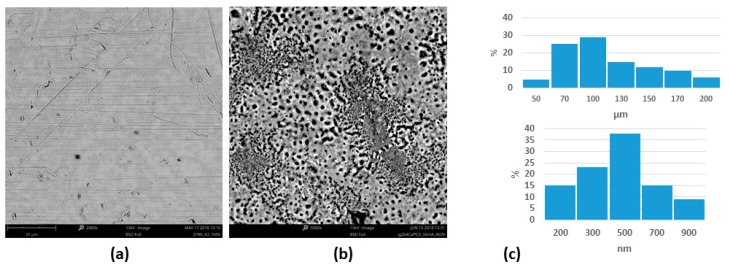
SEM image of the ZrNb alloy surface before (**a**) and after (**b**) the plasma electrolytic oxidation (PEO) process with pore distribution (**c**).

**Figure 3:**
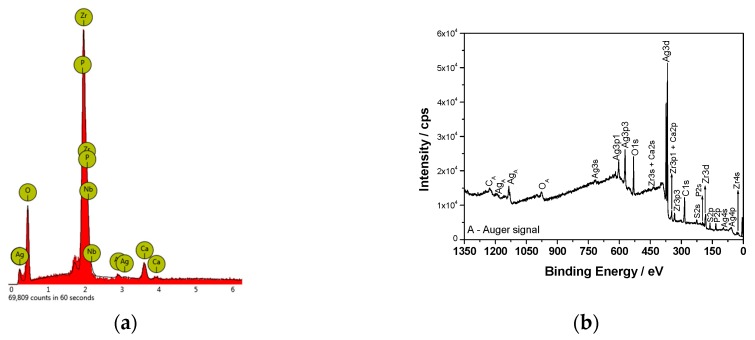
EDX (**a**) and XPS (**b**) spectra of the ZrNb alloy surface after the PEO process.

The AgNPs used in the experiment have a round shape with an average size of 27 ± 4.3 nm (Figure 1a). EDX analysis suggested the presence of pure silver with no presence of additional elements (Figure 1b). The surface of the ZrNb alloy used for the PEO process was rather smooth, with some rough areas remaining after polishing (Figure 2a). After the PEO process in electrolyte containing AgNPs, a mesoporous layer could be observed on the ZrNb alloy (Figure 2b) with pore sizes ranging from 200 nm to 70 µm. The pore size distribution shown in Figure 2c. The surface was not homogeneous, with patches of a PEO oxide layer with large pores and islands of submicro- and nanopores. There is some evidence that mesoporous structures can provide effective osteointegration and act as an antifouling surface for bacterial adhesion [13]. On the ZrNb alloy surface after the PEO process, silver particles are visible (light spots; Figure 2b). An EDX analysis (Figure 3a) confirmed the presence of Ca, P, and O in the ZrNb oxide layer. The silver peak is also clearly visible in the EDX spectra.

**Figure 4:**
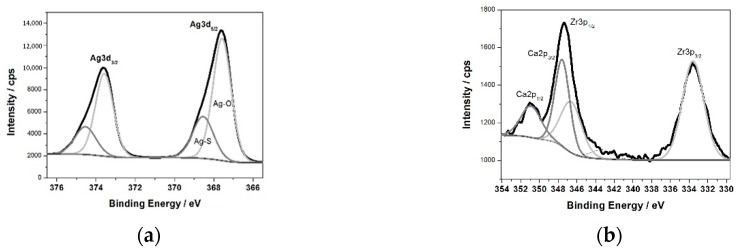
The XPS high-resolution spectrum of the Ag3d (**a**), Ca2p and Zr3p (**b**) peaks.

To evaluate the surface chemistry of the obtained coatings, XPS investigations were performed. The measured binding energies of the core electrons provide a large amount of information about the properties of the atoms in the solids. Figure 3b shows the XPS survey spectrum for the surface of the ZrNb alloy after plasma electrolytic oxidation in an electrolyte containing Ag. Characteristic XPS signals for particular elements have been identified, which are assigned to Ag3d, Ag3p, Ag3s, Ag4s, Ag4p, Zr3d, Zr3p, Zr4s, O1s, C1s, P2p, P2s, S2p, S2s, Ca2p, and Ca2s peaks and to Ag MNN, O KLL, and C KVV Auger peaks. Nb was not detected in the analysed layer, where the silver compounds are the main components: silver oxide (367.6 eV) and sulphide (368.5 eV). The high-resolution spectrum of the Ag3d peak after deconvolution is presented in Figure 4a, with spin-orbit separations of Δ = 6.0 eV. Further XPS analysis showed the presence of peaks located at 182.8 and 185.2 eV corresponding to Zr3d_5/2_ and Zr3d_3/2_ in ZrO_2_, respectively. The phosphorus P2p signals were attributed to phosphate groups, with a binding energy of 133.4 eV. In this case, the recorded signal for Ca2p coincides with the Zr3p_1/2_ peak. A careful analysis of the binding energy range from ~330 to ~355 led to determination of the Ca chemical state, as shown in Figure 4b.

The measured range was deconvoluted into four peaks: 333.6, 346.6, 347.6, and 350.9 eV. The first and second peak were related to Zr3p, with spin-orbit separations of Δ = 13.0 eV. The third and fourth signals were attributed to calcium phosphate compounds (Δ = 3.55 eV). The XPS data confirmed that PEO in an electrolyte containing Ag formed a coating consisting of a mixture of zirconium oxide and calcium phosphates enriched in silver compounds, namely, oxide and sulphide. Sulphur is probably a contamination. A chemical composition of the sample after PEO process is presented in Table 1. A high concentration of Ag in the oxide layer is worth of noting.

Contact angle of the ZrNb alloy after PEO process was equal 35° ± 10°. It means that the surface of the modified alloy is hydrophilic. The surface roughness was equal 1.6 ± 0.3 μm. Contact angle and roughness values can improve proliferation of cells on the ZrNb alloy surface, but also of bacteria.

**Figure 5:**
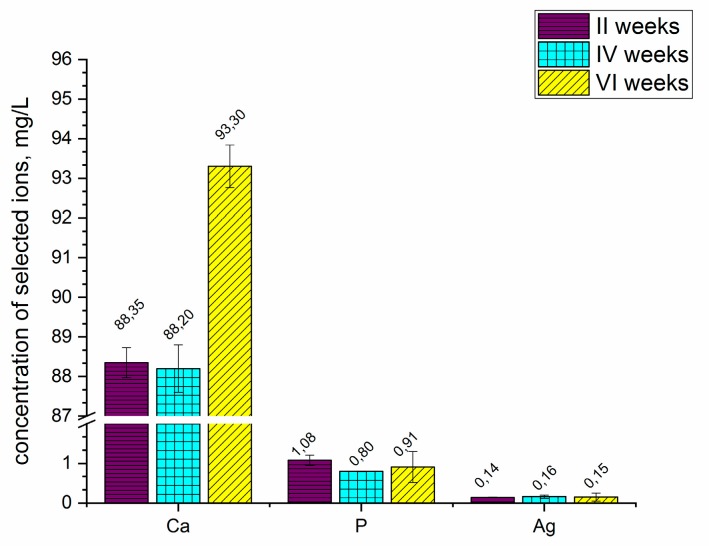
Concentration of selected ions released from the anodized ZrNb alloy into the Ringer solution. The concentrations of Zr and Nb were below 0.01 mg L^−1^.

Figure 5 presents the concentration of selected ions released from the coatings into the Ringer solution after 2, 4, and 6 weeks. The coating formed on the ZrNb alloy released the selected ions, such as calcium, phosphorous and silver, into the simulated body fluid (Ringer solution). Substrate-based ions such as niobium and zirconium were detectable; however, their concentration was below 0.01 mg L^−1^. Changes were observed in the concentration of calcium ions, especially after six weeks of coated sample immersion. The concentration of calcium significantly increased to 93.30 mg L^−1^ after that time. During 2 to 4 weeks of sample immersion, the concentration of calcium remained the same, as did the concentration of phosphorous and silver ions. The measurements showed that the coatings were able to degrade in the Ringer solution; however, the concentrations of phosphorous and silver ions were not significantly changed during the 6 weeks of immersion.

**Figure 6:**
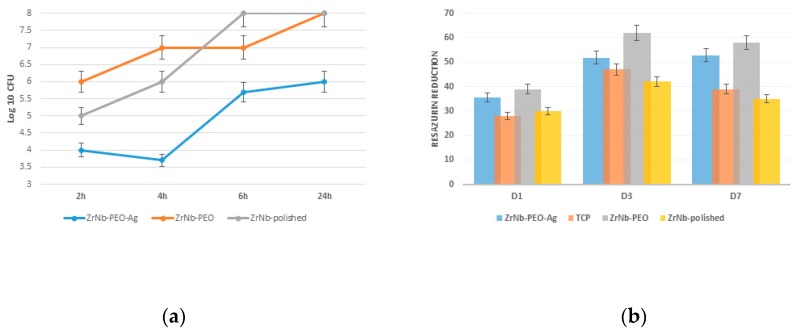
Bacterial adhesion over 24 h (**a**) and osteoblast adhesion and 7-day proliferation (**b**) on the PEO surface compared to that of the untreated polished control.

A bacterial study confirmed *S. aureus* adhesion and proliferation during the 24-hour experiment. After 2 h, the PEO-treated surface had 25% and 30% fewer adherent bacteria than that of the controls (polished and ZrNb-PEO treated sample), with a slight decrease in the microorganism level over 4 h (Figure 6a). The untreated and free of Ag PEO surfaces showed significant bacterial proliferation at all time points, reaching 8 Log 10 CFU after 6 and 24 h. The presence of AgNPs on the sample surface prevented bacterial growth, and the final *S. aureus* number did not exceed 6 Log CFU after 24 h. It is known that AgNPs can release Ag^+^ ions that disrupt bacterial function through reaction with the cell membrane [17]. Continuous ion release can protect the implant surface from bacterial contamination after surgery. To avoid bacterial contamination during long time cultivation, higher Ag concentration and continuous release within the time is required. But at the same time, it may provide toxic effects to attached cells that can decrease implant effectiveness. In our experiment, six bacteriostatic effects during 6 h can provide a “therapeutic window” for protein adsorption and osteoblast cell adhesion with future success at osteointegration.

There was no significant difference in osteoblast adhesion between the treated (with or without AgNPs) and untreated (polished) surfaces 1 d after cell seeding (Figure 6b). Significant (*p* ≤ 0.01) cell proliferation was observed on both surfaces after 3 d. The PEO Ag free surface showed significant cell proliferation compared to the ZrNb-PEO-Ag and polished surfaces. Seven days after cell seeding, the surface with and without AgNPs supported the same level of cells as that at day 3, but at the polished surface and TCP-control we observed a significantly lowered resazurin reduction level. This finding shows that compared to the untreated surface, the mesoporous surface provides a better environment for cell adhesion and proliferation and can be used as a base for enhanced osteointegration.

## 4. Conclusions

Plasma electrolytic oxidation of ZrNb alloy in a Ca-P based solution with AgNPs forms a mesoporous oxide layer that supports osteoblast cell adhesion and proliferation. Additionally, the obtained surface with incorporated Ag prevents bacterial adhesion in the first 6 h after immersion in a pathogen suspension, which can be an effective approach to prevent infectious complications after implantation.

## Figures and Tables

**Table 1:** The chemical composition of ZrNb alloy after the PEO process in AgNp solution.

Element	P	S	Zr	Ca	Ag	N	O	C
Concentration, at %	8.0	3.8	3.5	2.4	14.1	2.6	25.1	39.6
